# Hepatocyte growth factor (HGF) in fecal samples: rapid detection by surface plasmon resonance

**DOI:** 10.1186/1471-230X-5-13

**Published:** 2005-04-12

**Authors:** Fariba Nayeri, Daniel Aili, Tayeb Nayeri, Junyang Xu, Sven Almer, Ingemar Lundström, Britt Åkerlind, Bo Liedberg

**Affiliations:** 1 Divisions of Infectious Diseases, University Hospital, Linköping, Sweden; 2 Department of Physics and Measurement Technology, University of Linköping, Sweden; 3 Department of Gastroenterology & Hepatology, University Hospital, Linköping, Sweden; 4 Division of Clinical Microbiology, University Hospital, Linköping, Sweden

## Abstract

**Background:**

The development of biosensors, based on surface plasmon resonance (SPR) technology, enables monitoring of a variety of biospecific interactions without the need for chemical-, biological- or radiological-labelled reagents.

**Method:**

We utilised SPR to detect hepatocyte growth factor (HGF) in reconstituted faecal samples and studied samples from patients with infectious gastroenteritis (n = 20) and normal controls (n = 10). Mouse anti-human HGF monoclonal antibodies and recombinant human HGF receptor (c-Met)/Fc chimera were immobilised in flow cells of a CM5 biosensor chip.

**Results:**

We found that infectious gastroenteritis produced a higher signal response compared to controls, due to binding of HGF to monoclonal anti-HGF antibody as well as binding of HGF to c-Met receptor (p < 0.01). The SPR signal response correlated with results from ELISA (r = 72%, p > 0.001). The signal response decreased significantly (p < 0.05) when samples were diluted with dextran, because of reduction in both specific as well as unspecific binding of HGF to dextran. The decrease in the specific response might imply that the dextran- binding site for HGF overlaps with the antibody binding epitope, or that dextran binding induces a conformational change of the HGF molecule. Bands corresponding to HGF were found by gel electrophoresis of purified faeces in an affinity chromatography column immobilised by HGF ligands.

**Conclusion:**

Determination of HGF by SPR might be beneficial in diagnosis of acute situations that present with symptoms of gastroenteritis and may, possibly, guide appropriate medical treatments. This is to our knowledge the first report on the use of SPR for detection of HGF in faeces samples.

## Background

The SPR technique is suitable for studying biomolecular interactions on, or close to, a surface [1]. SPR enables rapid detection in real time without any labeling of samples and can be utilised for concentration determination, kinetics studies and epitope mapping. In brief, light incident on a metal surface at a given angle of incidence can excite a surface-bound electromagnetic wave, a surface plasmon, which propagates along the interface between the metal and the ambient medium. Associated with the surface plasmon is an evanescent field that probes local changes in the refractive index of the ambient medium, induced, for example, by binding of a biomolecule to the surface. The change in refractive index will shift the angle of incidence at which the SPR excitation occurs. This shift in the angle is tracked by monitoring the movement of intensity minima of the reflected light with time using the Kretschmann configuration, and the binding event is presented as a sensorgram [1]. The sensor surface of the SPR apparatus consists of a carboxy-methylated dextran matrix, which is a hydrogel providing a solution-like environment in which the biospecific interactions occur. The carboxyl groups in the dextran matrix enable covalent coupling of ligands (proteins, receptors, DNA etc) to the surface.

Hepatocyte growth factor (HGF) is a growth factor that is produced by mesenchymal cells during injuries in various organs. It stimulates cell division [2], motility [3], and a normal morphogenic structure [4] in the epithelial cells that have survived an injury (adjacent to the injured area). HGF is translated as a single-chain precursor and activated at the site of injury by proteolytic cleavage, resulting in a double-chained active HGF [5]. Interaction between the active HGF and its specific receptor (c-Met) [6] initiates intracellular signal pathways that result in regeneration and repair of damaged tissue [7]. High amounts of HGF have been determined systemically during injuries caused by infection [8]. At the site of infection, a local production of HGF has been found during bacterial meningitis and pneumonia [9,10].

Using a commercially available ELISA kit, we have shown that the concentration of HGF in faeces increases significantly during infectious gastroenteritis [11]. The levels decrease to normal after recovery. We have further studied the presence and stability of HGF in faeces samples during infectious processes [12]. Since traditional immunoassays (e.g., ELISA) are time-consuming and laborious, there is a need for quicker and simpler methods. In this study we present a method based on surface plasmon resonance, SPR, which allow us to detect and measure the levels of HGF in faeces in individual samples with the same accuracy but more rapidly than traditional methods.

## Methods

### Patients

Nine patients admitted to hospital with signs of acute untreated infectious diarrhoea were included. These patients had participated in a previous study [11]. Faeces samples were taken at admittance and cultured (*Campylobacter*, *Salmonella *and *Shigella*), and laboratory diagnostic tests for *Rotavirus *(antigen detection) and *Calicivirus *(electron microscopy) as well as cultures and cytotoxin analysis for *Clostridium difficile *were conducted. Cultures and cytotoxin analysis (toxin A) were positive for *Clostridium difficile *in 1 case. Cultures revealed growth of *Campylobacter jejuni *in 4 cases. *Salmonella *art (*Salmonella DO*) was found in 3 cases. Faeces culture was negative in one patient. This patient had diarrhoea and fever and his wife had gastroenteritis caused by *Campylobacter jejuni*. A second group of eleven patients with *Clostridium difficile *(positive culture and toxin A) were also analysed. This gave a total of 20 samples of faeces from 20 patients (11 women, 9 men, range 20–85, median 51.5 and mean 52 year). Faeces haemoglobin (Actim Fecal Blood test; Orion Diagnostica) was examined and was positive in 10/13 samples.

### Controls

Faeces samples were obtained from 10 healthy vaccination volunteers (six women and four men, 20–60 years) without signs of infection or diarrhoea. Faeces culture and diagnostic tests for Rotavirus and Caliciviruswere negative in all of these cases. These patients had negative Actim Fecal Blood test results (Orion Diagnostica)

### Standardizing faeces volume and reconstitution procedure

All faeces samples were stored at -20°C. Prior to handling, the samples were thawed at room temperature and mixed using a Vortex (Vortex-Genie, Scientific Industries Inc., Bohemia, NY, USA). The samples were then placed at -70°C for 15 minutes, followed by room temperature for 2 minutes and dissolved in distilled water at a dilution of 1:6. The suspension was centrifuged at 1000–3000 *G *for 15 minutes and the supernatant stored at -70°C until analysed.

To avoid the effects of digestive as well as bacterial enzymes, protease inhibitor (1–5%) (Sigma Aldrich) with specific inhibition of serine, cysteine, aspartic proteases and aminopeptidases, containing 4-(2-aminoethyl) benzenesulfonyl fluoride (AEBSF), pepstatinA, E-64, bestatin, leupeptin, and aprotinin (but no metal chelators) was added to the thawed samples (room temperature) 30 minutes prior to analysis.

### SPR measurements and ligand immobilisation procedures

SPR measurements were conducted at 760 nm in a fully automatic Biacore 2000 instrument and a semi-automatic Bicaore X instrument (Biacore AB, Uppsala, Sweden) equipped with four and two flow cells respectively. The flow cell temperature was 25°C in all experiments. The sample surfaces used were carboxy-methylated dextran CM5 chips (Biacore AB, Uppsala, Sweden). Coupling of ligands to the carboxylic acid groups of the dextran hydrogel was carried out by conventional carbodiimide chemistry using 200 mM EDC (N-ethyl-N' -(3-diethylaminopropyl) carbodiimide) and 50 mM NHS (N-hydroxysuccinimide). The activation time was 7 min, followed by a 2–7 min ligand injection. Deactivation of remaining active esters was performed by a 7 min injection of ethanolamine/hydrochloride at pH 8.5. A flow rate of 5 μl/min was used during immobilisation. All ligands were diluted in 10 mM acetate buffer pH 4.5, i.e., below the protein isoelectric point, thus enhancing the electrostatic interactions between the dextran matrix and the ligands. The monoclonal anti-HGF (500 μg/ml) was diluted 1:10, the recombinant Met proto-oncogene receptor (100 μg/ml) 1:5, and the HGF recombinant (5 ug/ml) 1:3. The contact time varied between two and seven minutes resulting in levels of immobilisation between 8000 and 30000 RU (response units). After deactivation, the surfaces were washed with five subsequent one-minute injections of 5 mM glycine buffer pH 2.0 with 1 M NaCl. One of the flow cells was used as a reference to monitor the response due to buffer and unspecific interactions. This flow cell was treated in the same way as the other during the immobilisation procedure, but omitting the ligand immobilisation step.

### Determination of faeces HGF by ELISA

After storage all samples were thawed and centrifuged at 1000 *G *for 15 minutes prior to analysis. Immunoreactive HGF was determined by ELISA using a commercially available kit (Quantikine HGF Immunoassay, R&D Systems Inc., Minneapolis, USA).

### Determination of faeces haemoglobin

Actim Fecal Blood (Orion Diagnostica) is an immunochromography technique that is specific for the determination of human haemoglobin using two monoclonal antibodies. The detection limit in faeces is 50 μg haemoglobin/L or 25–50 μg haemoglobin/g. The method detects the intact haemoglobin molecule, but neither haemoglobin that is influenced by the enzymes during gastrointestinal passage nor animal haemoglobin is detected.

### Purification of faecal samples by affinity chromatography, ultrafiltration, and SDS-PAGE

The affinity chromatography columns (Hi-trap Amersham Biosciences) were immobilised with monoclonal anti-human HGF and recombinant human HGF receptor (c-MET)/Fc chimera (R&D Systems) respectively. Faeces samples were reconstituted in distilled water. The samples were applied to the 500 μl centrifugal tubes (Amicon Ultra, Millipore, S.A.S. Molsheim, France) and centrifuged at 4000 G for one hour. SDS-PAGE was performed with the faeces samples before and after purification and filtration, using a 4% stacking gel and a 12% running acrylamide gel [13]. For size estimation prestained protein standard (Sigma Aldrich) was electrophoresed simultaneously.

### Statistics

Non-parametric Friedman and Wilcoxon signed rank tests (absolute values, Statview & SPSS Base 11.0) were used. The Spearman rank correlation coefficient was used for analysis of correlation between parameters. A p-value ≤ 0.05 was regarded as statistically significant.

## Results

**Figure 1:**
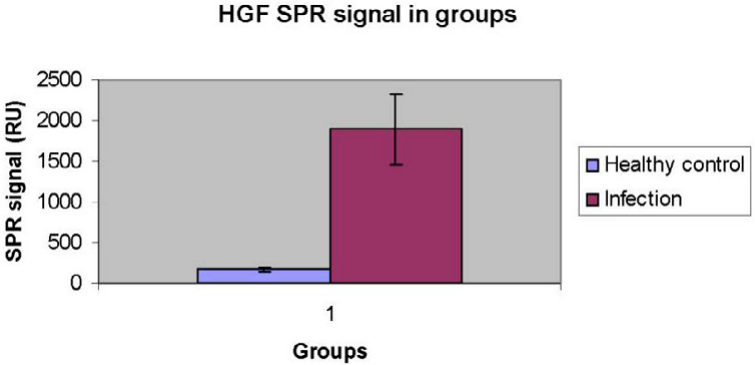
Histogram showing signal responses determined by SPR (channel immobilised by mouse anti-human HGF monoclonal antibodies) in infectious gastroenteritis (n = 20, median 1895 ± 429 RU, range 123–6660 RU) and healthy controls (n = 10, median 171 ± 38 RU, range 14–459 RU). The response is defined as the difference in response signal before and after the injection of the sample minus the difference observed in the reference channel.

**Figure 2:**
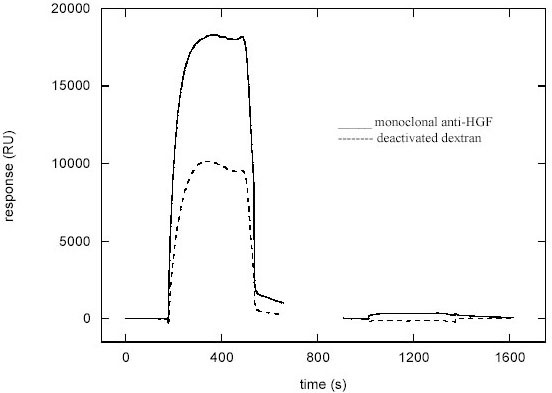
SPR sensogram showing the signal obtained from analysis of faeces from a patient with infectious gastroenteritis. Note the difference in signal intensity between the control channel (dextran) and the channel immobilised by mouse anti-human HGF monoclonal antibody.

Patients with infectious gastroenteritis had significantly higher signal responses in flow cells immobilised with monoclonal anti-HGF antibody compared to the healthy controls (determined in at least six independent experiments)(Friedman p = 0.006, Wilcoxen Signed Rank Tests p = 0.008) (Fig 1 and 2). The signal responses in the flow cells immobilised with c-Met receptor were also significantly higher in the group with infectious gastroenteritis (Wilcoxen Signed Rank Tests, p = 0.02).

### Correlation with ELISA

**Figure 3:**
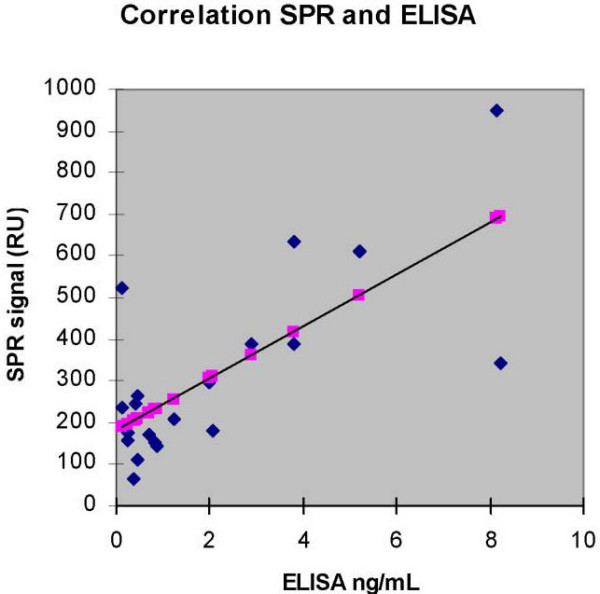
Correlation between the concentration of HGF in faeces obtained by ELISA and signal responses by SPR (Spearman rank correlation coefficient n = 20, r = 0.72, p < 0.001). The shadow points along the regression line are not data points.

Determination of HGF in faeces (patients with infectious gastroenteritis) by utilising SPR correlated significantly (n = 20, r = 72%, p < 0.001) with the levels measured by ELISA (range 0.14–8.24, median 0.83 ng/mL) (Fig 3). HGF levels determined by ELISA in healthy controls (n = 10) were low (range 0.01–0.26, median 0.06)

### Correlation with age

**Figure 4:**
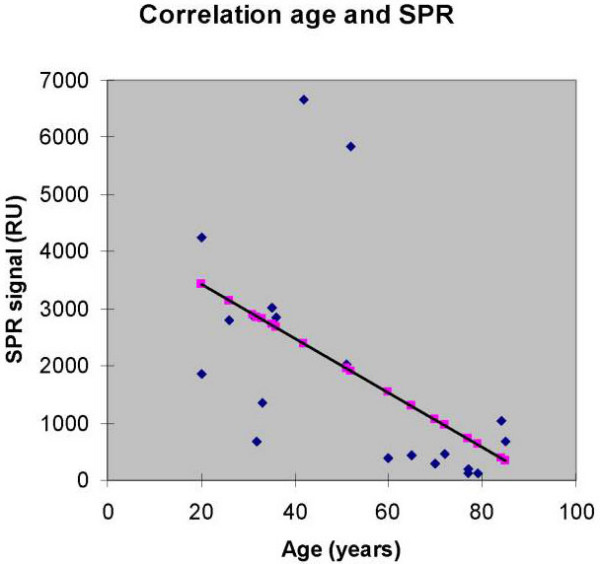
Correlation between age of the patients with infectious gastroenteritis and signal responses by SPR (Spearman rank correlation coefficient r = - 0.55, p = 0.01). The shadow points along the regression line are not data points.

We found a negative correlation between the SPR signal intensity and age in patients with infectious gastroenteritis (n = 20, r = - 0.55, p = 0.010) (Fig 4). The patients with acute gastroenteritis were older than the healthy controls (median 51.5 respective 35.0 years).

### Effects of altering the immobilisation levels

**Figure 5:**
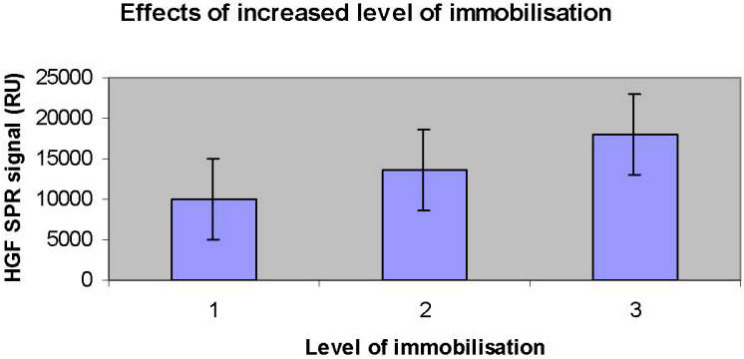
Histogram showing the effects of increasing the immobilisation levels by increasing the contact time during immobilisation (1–3 representing 1, 5, and 10 minutes) (Friedman p < 0.01, Wilcoxon signed ranks test between 1 and 5 minutes p = 0.058, between 5 and 10 minutes p = 0.003, and between 1 and 10 minutes p = 0.003). The monoclonal anti-HGF (500 μg/ml) was diluted 1:10 in 10 mM acetate buffer pH 4.5. The activation time was 7 min, followed by a 1,5 and 10 min ligand injection respectively. Deactivation of remaining active esters was performed by a 7 min injection of ethanolamine/hydrochloride at pH 8.5. A flow rate of 5 μl/min was used during immobilisation.

The effect on signal responses due to the amount of monoclonal anti-HGF antibody immobilised was determined in different samples (n = 12). Increasing the immobilisation levels by increasing the contact time during immobilisation (1, 5, and 10 minutes) caused a significantly higher response of all samples (Friedman p < 0.01, Wilcoxon signed ranks test between 1 and 5 minutes p = 0.058, between 5 and 10 minutes p = 0.003, and between 1 and 10 minutes p = 0.003) (Fig 5). Altering the flow rate (5, 10, and 15 μl/min) did not have a significant effect on the response of the different samples (Friedman p = 0.48) (data not shown). When altering levels of immobilisation of the c-Met receptor (n = 15), no effect in responses in the case of infection (P = 0.85) was observed. Changing of the flow rate (5, 10, and 15 μl/min) did not influence the signal responses (P = 0.36) (data not shown).

### Binding of HGF to dextran

Addition of dextran (0.05%) to the samples resulted in a significant (p < 0.05) decrease in signal responses in flow cells with immobilised anti-HGF but did not affect the HGF binding to c-met (p = 0.11). Diluting recombinant HGF with dextran (0.05%) also resulted in a decrease of binding to the monoclonal anti-HGF antibody (n = 3). The test of significance was not performed.

### SDS-PAGE

**Figure 6:**
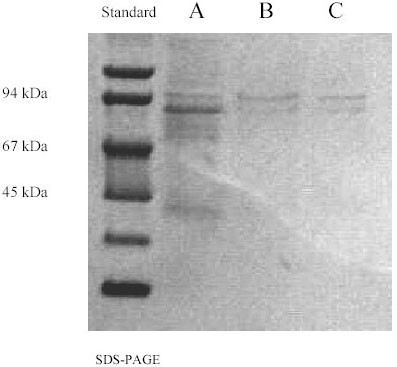
SPS-PAGE of faeces. A: before purification; B: after purification in an affinity chromatography column immobilised by mouse anti-human HGF monoclonal antibody; C: after purification in an affinity chromatography column immobilised by recombinant human c-met/fc chimera.

We investigated the presence of HGF in the samples by SDS-PAGE of faeces samples as well as SDS-PAGE of purified faeces samples in an affinity chromatography column immobilised by either mouse anti-human HGF monoclonal antibody or recombinant human c-met receptor. Bands corresponding to the HGF were detected with apparent molecular masses of 75–90 kDa (Fig 6).

### Protease Inhibitor

**Figure 7:**
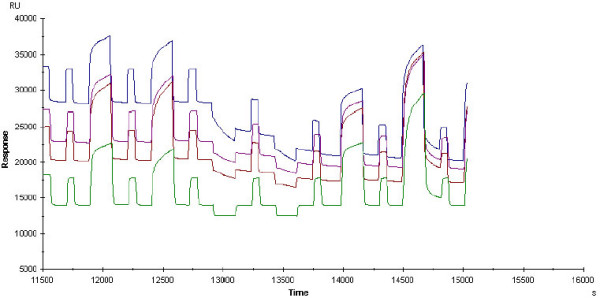
SPR sensogram from faeces analysis. The flow cells were immobilised by recombinant Met proto-oncogene receptor (20 μg/ml), monoclonal anti-HGF (50 μg/ml) and HGF recombinant (3 μg/ml), respectively. The last channel (lowest line) was used as a reference to monitor the response due to buffer and unspecific interactions. Every other signal is the regeneration signal (1:1 combination of 1M Nacl and Glycine pH 2.0). The signals at time limits 12000, 12500, 14100 and 14600 seconds belong to patients with infectious gastroenteritis. The signals at time limits 13000 and 13500 seconds belong to faeces from healthy control. Please note the decline in the base line of the flow cells at the time limit 13000 when a faeces sample was injected that did not contain protease inhibitor.

Faeces destroyed the proteins immobilised in the flow cells of the CM5 chip (Fig 7). Adding protease inhibitor (1–5%) to the faeces samples within 30 minutes prior to experiments could inhibit this effect completely.

## Discussion

In the present work the Surface Plasmon Resonance (SPR) based method has been used for the first time to evaluate faecal samples and monitor the relative amount of HGF in the samples.

HGF has been investigated widely over the past decade. The unique properties of this cytokine, makes it likely to be involved in the recovery process after injuries [3]. Recent studies have strengthened the notion that HGF plays an important role in the regeneration of an injured organ [2]. Several researchers have attempted to treat induced hazardous injuries in animal models by HGF [14,15]. We have studied HGF during infectious diseases and found that HGF was produced in high amounts both systemically and locally during injuries caused by infection [8]. Low amounts of serum HGF in patients with pneumonia correlated significantly to poor prognosis [16]. Application of HGF locally at the site of an injury such as a chronic ulcer resulted in an accelerated healing process [17].

The gastrointestinal mucosa has a remarkable ability to repair damage, and growth factors play an important role in the regeneration of injured cells in gastrointestinal organs [18]. Nishimura et al. (1998) [19] showed that HGF was the most potent of the cytokines (HGF, TGF-α, TGF-β, and keratinocyte growth factor) in accelerating repair of the damaged monolayer of an epithelial cell line derived from normal rat small intestine. We studied the amounts of HGF in faeces of patients with infectious gastroenteritis and found that the levels of HGF were significantly elevated during infection [11]. Although this observation might indicate healing of injury caused by an infection, we could discriminate with high specificity and sensitivity between infectious gastroenteritis and other disorders that present with diarrhoea [11].

In a previous study [12] the concentration and stability of HGF in fecal samples was investigated using ELISA. Although the ELISA method was very reliable, it was laborious and gave no information about the form of HGF that was present in the samples. In the present study we found that there is good correlation between the results obtained from SPR and ELISA measurements of faecal HGF levels. In some cases differences between results obtained by ELISA and SPR are found and this phenomenon is to be further investigated.

Using SPR we were able to monitor the presence of HGF in the samples and the interactions between HGF and different ligands with different binding specificity to the HGF molecule. First, a recombinant HGF that showed affinity to the monoclonal anti-HGF antibody as well as c-met receptor was examined (data not shown). The affinity of recombinant HGF disappeared within one week after reconstitution of the lyophilised form (data not shown). On the other hand, the signal responses after analysis of faeces samples in the patients with infectious gastroenteritis were stable after reconstitution. We have tested widely the stability of HGF in faeces samples by ELISA [12] and previously in serum [20]. Unlike our previous observations [11] that showed no significant correlation between age of patients and HGF levels determined by ELISA, in this study we found a negative correlation between age and SPR signal in the group with infectious gastroenteritis. This might strengthen the notion that the ability of HGF to interact with its ligand or possibly its activity might change by age. Moreover, in spite of the fact that patients with infectious gastroenteritis were older than healthy controls they had higher SPR signals.

Presence of blood was shown in ten out of thirteen cases with infectious gastroenteritis while it was negative in healthy controls. However we have previously studied the correlation between presence of blood in faeces and HGF levels (determined by ELISA) and did not find any significant correlation [12].

The immoblised layer in the flow cells of the chip were destroyed after analysis of faeces samples (Fig 7). Adding protease inhibitor to the samples before analysis inhibited this effect which might indicate the presence of proteases in faeces. We have previously shown that adding protease inhibitor to faeces does not influence the ability to detect HGF by ELISA [12].

At least two binding sites for HGF on liver cell surfaces have been demonstrated in rat: the heparin-resistant and acid-washable site (HGF receptor, binding site) and the heparin-washable site (low-affinity, cell-surface heparan sulfate proteoglycan binding site) [21]. HGF has also been reported to interact with the extracellular matrix [22]. The low affinity sites may serve as a reservoir for the regulation of endogenous HGF levels and provide a matrix for conversion of promitogen to the two-chain form [21]. The HGF detected during infectious gastroenteritis had binding affinity to dextran (proteoglycan). By adding carboxy-methyl dextran (0.05%) to the samples, the levels of HGF bound to the immobilised monoclonal anti-HGF decreased significantly. Adding the same amount of dextran to recombinant HGF gave the same result in signal responses (data not shown). This might indicate a binding affinity of this form of HGF to dextran, which might resemble such affinity to cell-surface heparan sulfate proteoglycan binding sites. These binding sites either overlap with the antibody-binding epitope or induce conformation shift in the HGF upon binding to the dextran, resulting in sterical hindrance and thus preventing binding to the antibody.

## Conclusion

Infectious gastroenteritis is a common disease in all societies. Determination of HGF by SPR is a reliable method to evaluate the relative amounts of HGF in faeces. This might be beneficial in diagnosis of acute situations that present with symptoms of gastroenteritis and may, possibly, guide appropriate medical treatments.

## Competing interests

The corresponding author is applying a patent that might be indirectly connected to the manuscript:

-Rapid determination of hepatocyte growth factor (HGF) in the body fluids.

There are no other financial competing interests.

## Authors' contributions

**1- FN: **Participated in design of method, design of study and collecting of samples and drafted the manuscript.

**2- DI: **Participated in the design of method and in performing the statistical analysis.

**3- TN: **Performed the reconstitution procedure of faeces samples. Participated in design of method. Performed the SPR analysis.

**4- JX: **Carried out the affinity chromatography and SDS-PAGE studies for HGF in faeces.

**5- SA: **Participated in design of study, collecting of samples and statistical analysis.

**6- BÅ: **Performed the microbiological assessment of samples.

**7- IL: **Participated in design and co-ordination of the study

**8- BL**: Participated in design and co-ordination of the study and evaluation of reliability of the new method as an expert.

All authors read and approved the final manuscript.

## Pre-publication history

The pre-publication history for this paper can be accessed here:
